# Switching from Twice-Daily Basal Insulin Injections to Once-Daily Insulin Degludec Injection for Basal-Bolus Insulin Regimen in Japanese Patients with Type 1 Diabetes: A Pilot Study

**DOI:** 10.1155/2015/176261

**Published:** 2015-09-07

**Authors:** Yuka Tosaka, Akio Kanazawa, Fuki Ikeda, Mayu Iida, Junko Sato, Kazuhisa Matsumoto, Toyoyoshi Uchida, Yoshifumi Tamura, Takeshi Ogihara, Tomoya Mita, Tomoaki Shimizu, Hiromasa Goto, Chie Ohmura, Yoshio Fujitani, Hirotaka Watada

**Affiliations:** ^1^Department of Metabolism & Endocrinology, Juntendo University Graduate School of Medicine, 2-1-1 Hongo, Bunkyou-ku, Tokyo 113-8421, Japan; ^2^Center for Therapeutic Innovations in Diabetes, Juntendo University Graduate School of Medicine, 2-1-1 Hongo, Bunkyou-ku, Tokyo 113-8421, Japan; ^3^Sportology Center, Juntendo University Graduate School of Medicine, 2-1-1 Hongo, Bunkyou-ku, Tokyo 113-8421, Japan; ^4^Center for Molecular Diabetology, Juntendo University Graduate School of Medicine, 2-1-1 Hongo, Bunkyou-ku, Tokyo 113-8421, Japan

## Abstract

The aim of this study was to investigate the efficacy of insulin degludec used for basal-bolus insulin regimen after switching from twice-daily basal insulin in Japanese patients with type 1 diabetes mellitus. The subjects were 22 type 1 diabetes patients treated with basal-bolus insulin regimen with twice-daily basal insulin. Basal insulin was switched to once-daily injection of insulin degludec with 10% dose reduction. HbA1c and fasting plasma glucose (FPG) were measured before and 12 weeks after switching. The frequency of hypoglycemic episodes, standard deviation (SD) of blood glucose, and mean of daily difference (MODD) were evaluated by continuous glucose monitoring (CGM) before and 4 weeks after switching. HbA1c and FPG before and 12 weeks after switching were comparable (HbA1c 8.5 ± 1.4 versus 8.7 ± 1.6%, *P* = 0.28; FPG 203.2 ± 81.2 versus 206.5 ± 122.4 mg/dL, *P* = 0.91). The frequency of hypoglycemia during nighttime was not significantly different at 4 weeks after switching (14.4 ± 17.0 versus 11.1 ± 15.0%, *P* = 0.45). In addition, SD and MODD before and 4 weeks after switching were also comparable. In conclusion, glycemic control under once-daily insulin degludec injection was almost comparable to that under twice-daily basal insulin injections in Japanese type 1 diabetes patients. This study was registered with ID: UMIN000010474.

## 1. Introduction

Intensive insulin therapy using basal-bolus insulin regimen is the standard therapy for patients with type 1 diabetes mellitus. By mimicking the endogenous insulin secretion profile in healthy subjects, it has been shown to improve glycemic control and reduce the risk of long-term complications compared with conventional insulin therapy [1, 2]. Unfortunately, many patients with type 1 diabetes cannot achieve the target glycemic control, and insulin therapy leaves room for improvement. Thus, the efficacy of basal insulin is especially important in this group of patients. Importantly, in some type 1 diabetes patients with severe loss of endogenous insulin secretion capacity, once-daily injection of basal insulin does not always cover the basal effect of insulin over the 24-hour period [3–5]. Hence, a second supplementary basal insulin injection is often used in these patients. In addition, the intraday and day-to-day variability in insulin agents could sometimes be an obstacle for optimized titration of insulin and a cause of increased frequency of hypoglycemia. Given that increased frequency of injection and large fluctuations in blood glucose could be a burden in such patients, any improvement in the efficacy of basal insulin agents should be appreciated.

Insulin degludec is a new ultra-long-acting basal insulin that forms soluble multihexamers at the subcutaneous injection site from which insulin monomers are slowly and continuously absorbed into the circulation, leading to a peakless action profile over 42 hours [6]. Consistent with this pharmacological action, BEGIN Basal-Bolus Type 1 Trial [7] showed that the rate of nocturnal-confirmed hypoglycemia was 25% lower with insulin degludec than with insulin glargine. In addition, it was reported that the day-to-day variability in plasma glucose in type 1 diabetes patients treated with insulin degludec was lower compared to insulin glargine [8]. Taking these unique actions of insulin degludec into consideration, switching from twice-daily injections of basal insulin to once-daily injection of insulin degludec could provide great benefit to patients with type 1 diabetes.

In this study, to evaluate the efficacy of insulin degludec as a basal insulin for basal-bolus regimen for Japanese patients with type 1 diabetes mellitus who are treated with twice-daily basal insulin injection therapy, we investigated glycemic control, daily, and day-to-day variability in plasma glucose using continuous glucose monitoring before and after switching to once-daily insulin degludec injection in 22 patients with type 1 diabetes.

## 2. Subjects and Methods

### 2.1. Subjects

We recruited 24 eligible Japanese patients (8 males and 16 females) with type 1 diabetes who visited the outpatient clinic of Juntendo University Hospital between July 2013 and January 2014. Patients who satisfied the following conditions were included: (1) treated with basal-bolus insulin regimen with twice-daily injections by insulin glargine or detemir and (2) aged more than 20 and less than 80 years. Also, patients were excluded if they (1) had serious liver disease (AST and/or ALT >100 IU/L), (2) had serious kidney disease (serum creatinine >2.0 mg/dL), (3) had untreated severe diabetic retinopathy, (4) had adrenal or pituitary insufficiency, (5) had other conditions considered by the attending physician to be contraindicated to inclusion in the study, or (6) were pregnant or breastfeeding women.

This trial was conducted in accordance with the Declaration of Helsinki, and the protocol was approved by the Human Ethics Committee of Juntendo University. All patients provided a written informed consent prior to trial initiation.

### 2.2. Study Design

**Figure 1:**
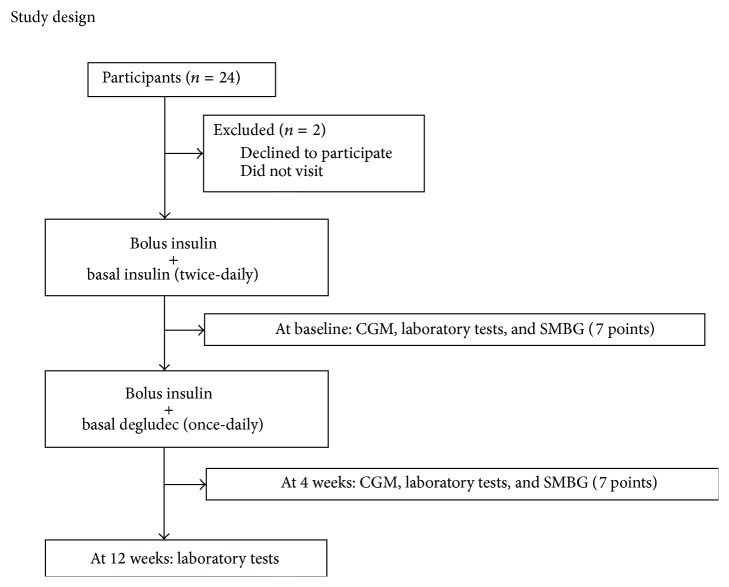
Study design. CGM was performed at baseline and 4 weeks after switching to insulin degludec. At 12 weeks after switching, glycemic control was evaluated by HbA1c, glycosylated albumin, and fasting plasma glucose.

In this prospective, single-center, single-arm, open-label, 12-week study, we compared the effects of switching from twice-daily basal insulin to once-daily insulin degludec on glycemic control, daily, and day-to-day variability in plasma glucose.  Figure 1 shows the patient enrolment process. At baseline before switching, the following laboratory tests were performed in each patient: fasting plasma glucose (FPG), plasma C-peptide, HbA1c, and glycosylated albumin. Plasma C-peptide assay was performed using ultrasensitive C-peptide ELISA kit (Mercodia, Uppsala, Sweden) for precise determination of intrinsic basal insulin level [9]. Then, CGM and 7-point self-measured blood glucose (SMBG) profiles (before and 2 hours after meals and bedtime) were obtained. After that, the patient was switched from twice-daily basal insulin to once-daily insulin degludec, which involved 10% reduction in insulin dosage without any change in the rapid acting insulin therapy. Insulin degludec was administered once-daily at bedtime. At 4 weeks after switching, the same fasting laboratory tests, CGM and 7-point SMBG, were repeated. After 4 weeks, the basal insulin dose was adjusted for each individual patient based on self-measured FBG levels taken before breakfast. The dose of insulin degludec was decreased by 1 unit if FBG was ≤ 80 mg/dL over three consecutive days just before the hospital visit. Then, the increase of the dose of basal insulin or titration of rapid acting insulin was performed by the judgement of each physician in charge. At the end of the study (12 weeks), the same laboratory tests (FPG, HbA1c, and glycosylated albumin) were repeated again.

### 2.3. Continuous Glucose Monitoring

CGM data were obtained by using the iPro2 (Medtronic; Northridge, CA). Patients were required to use CGM for six consecutive days. Over each CGM occasion, at least 288/day CGM glucose values were to be recorded. As an index of day-to-day variability, the mean of daily difference (MODD) was calculated from the absolute difference between paired CGM values during two successive days (days 2 to 3 and days 4 to 5), and the data were presented as the average of the two values. The patient was asked to record 7-point SMBG profiles for one day during CGM for before and 2 hr after meals and at bedtime.

The primary outcome of the study was change in HbA1c before and 12 weeks after switching. The secondary outcomes based on CGM values were (1) changes in standard deviation (SD) and MODD [10]. Safety variables included the frequencies of severe hypoglycemia, which was defined as low blood glucose level requiring assistance from another person to treat, nocturnal hypoglycemia, and adverse events. Confirmed hypoglycemia was defined as a glucose value of less than 70 mg/dL by CGM and was reported in percentage (= times <70 mg/dL/total time of measurement). Hypoglycemic episodes occurring between 0:00 and 5:59 hours were classified as nighttime while daytime episodes occurred between 6:00 and 23:59. Safety assessment included hypoglycemic events by CGM and adverse events by laboratory tests.

### 2.4. Statistical Analysis

Data were expressed as mean ± SD. The Mann-Whitney *U* test was used for analysis of CGM data before and 4 weeks after switching and, for analysis of FPG, HbA1c and glycosylated albumin before and 12 weeks after switching were used. A *P* value < 0.05 was considered statistically significant. All statistical analyses were conducted using StatView statistical software package, version 5.0 (SAS Institute Inc., Cary, NC).

## 3. Results

### 3.1. Baseline Characteristics of the Subjects

Two patients withdrew from the study after the first treatment period; one decided to withdraw during the conduct of the study and the other did not visit the outpatient clinic. The full analysis was conducted in the remaining 22 patients. The clinical characteristics of the patients are shown in Table 1. The mean age and duration of type 1 diabetes mellitus were 54.8 ± 14.5 and 14.6 ± 9.0 years, respectively. Fasting plasma C-peptide was below the detection limit of the ultrasensitive C-peptide ELISA kit in 18 patients (81%), indicating severely low insulin secretion in most subjects.

### 3.2. Effects of Switching to Insulin Degludec on Glycemic Control

As shown in Table 2, HbA1c levels at baseline and 4 and 8 weeks after switching to insulin degludec were 8.5 ± 1.4%, 8.6 ± 1.6%, and 8.7 ± 1.6%, respectively. Glycosylated albumin levels before and 4 and 8 weeks after switching were 24.9 ± 5.0%, 25.3 ± 5.2%, and 24.7 ± 3.6%, respectively. Furthermore, fasting blood glucose levels were 203.2 ± 81.2 mg/dL, 165.5 ± 82.1 mg/dL, and 206.5 ± 122.4 mg/dL, respectively. Based on these data, it is clear that switching to insulin degludec did not improve glycemic control throughout the study. The mean basal insulin and total daily doses at 12 weeks after switching to insulin degludec were significantly reduced compared to the baseline (15.2 ± 7.6 versus 11.6 ± 6.9 U, *P* < 0.01, and 40.0 ± 17.3 versus 37.9 ± 16.7 U, *P* < 0.01, resp.) whereas the bolus insulin dose did not significantly change after switching.

### 3.3. Effects of Switching to Insulin Degludec on Glucose Fluctuation and the Frequency of Hypoglycemia

Table 3 summarizes fluctuations in glucose level and the frequency of hypoglycemia recorded by CGM over 4 days before and after switching. The averages of blood glucose and standard deviation (SD) through the daytime (0:00–23:59) were 184.1 ± 45.8 versus 189.6 ± 52.7 mg/dL and 68.4 ± 14.5 versus 66.5 ± 17.1 mg/dL, respectively. Furthermore, the MODD was 72.1 ± 16.0 versus 74.0 ± 23.0 mg/dL and the frequency of hypoglycemia below 70 mg/dL was 6.1 ± 8.0 versus 6.5 ± 9.7%, respectively. These data indicate no significant changes in these parameters after switching.

Table 3 also shows fluctuations in glucose level and the frequency of hypoglycemic episodes recorded by CGM during the nighttime (0:00–5:59). The averages of blood glucose before and after switching were 156.5 ± 63.7 versus 174.6 ± 58.5 mg/dL during the nighttime. The SD during nighttime did not change significantly (before: 22.6 ± 10.9, after: 24.8 ± 10.7 mg/dL). These data indicate no significant changes in these parameters after switching to insulin degludec. MODD tended to increase during the nighttime both at baseline and after switching, however; these changes were not significantly different. The frequency of hypoglycemic glucose (below 70 mg/dL) was 14.4 ± 17.0 versus 11.1 ± 15.0% during the nighttime, before and after switching. Thus, the frequency of nocturnal hypoglycemia did not change significantly after switching, similar to other parameters, and no severe hypoglycemia was recorded during the study period.

### 3.4. 7-Point SMBG Profiles before and after Switching to Insulin Degludec

**Figure 2:**
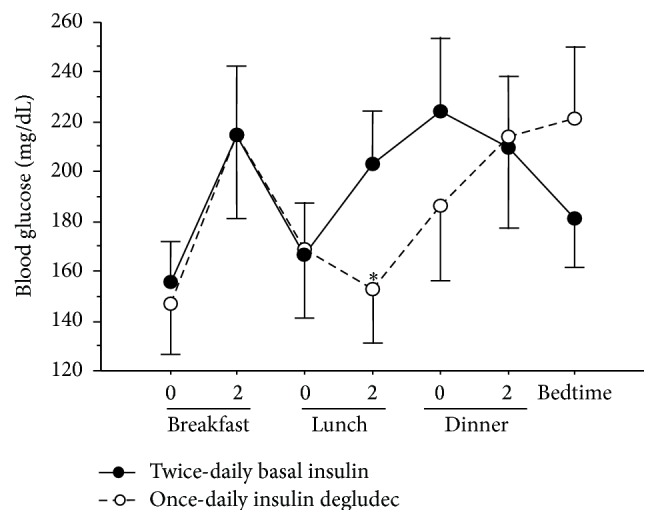
Average daily profile of blood glucose in patients treated with twice-daily basal insulin and once-daily insulin degludec. 0 and 2: blood glucose level before and 2 hours after the indicated meal. Blood glucose levels at 2 hours after lunch were significantly lower after switching than before switching. ^*∗*^ *P* < 0.01 versus twice-daily basal insulin.

Figure 2 shows the 7-point SMBG profiles before and 4 weeks after switching. Blood glucose level at 2 hours after lunch was significantly low before and after switching (207.6 ± 87.7 versus 158.2 ± 90.3 mg/dL, *P* < 0.01). No significant changes were noted in all other parameters derived from blood glucose levels.

## 4. Discussion

The present study investigated the efficacy and safety of switching from twice-daily basal insulin injections to once-daily insulin degludec injection in Japanese patients with type 1 diabetes. Insulin glargine and detemir are often used as basal insulin in daily clinical practice. However, some patients with extremely low insulin secretion capacity often need to use twice-daily basal insulin because the duration of action of insulin detemir is about 16 hours [11] and that of insulin glargine does not last up to 24 hours [4, 12].

A few studies from Japan have already investigated the outcome of switching from once-daily or twice-daily basal insulin to once-daily insulin degludec in patients with type 1 diabetes [13–15]. Specifically, these studies investigated the effects of switching to insulin degludec in type 1 diabetes patients who were being treated with a combination of once- or twice-daily injections of insulin glargine or detemir, though there are no studies that focused on type 1 diabetes patients treated only with twice-daily basal insulin.

The basal insulin levels were very low in our patients and the level could not be detected in most patients even by using high-sensitivity C-peptide kits with detection limit of <0.015 ng/mL. These results indicate that the subjects were insulin-dependent. Therefore, the selection of twice-daily basal insulin injections in our study seems reasonable to achieve better glycemic control. Insulin degludec, an ultra-long-acting basal insulin, became available in Japan in 2013, ahead of other countries, and is known to have longer duration of action (over 42 hours) compared with insulin glargine and detemir. Therefore, it is clinically worthy to investigate the efficacy and safety of switching from twice-daily insulin glargine or detemir to once-daily insulin degludec in type 1 diabetes patients with severely reduced insulin secretion. The results showed no significant changes in various parameters of glycemic control, such as fasting plasma glucose, HbA1c, and glycosylated albumin, after switching to insulin degludec despite about 20% reduction in basal insulin dose at 12 weeks, indicating that insulin degludec has longer duration of action and a more potent glucose-lowering effect than insulin glargine or insulin detemir.

The frequency of hypoglycemic episodes recorded by CGM did not increase at 4 weeks after switching to insulin degludec. In our study protocol, the bolus insulin dose was not changed before and after CGM recording because the effect of bolus insulin on glycemic control needed to be minimized. According to a previous report by the BEGIN Basal-Bolus Type 1 Trial investigators [7], the mean doses of basal and premeal bolus insulin were significantly decreased by 14% and 10% in the insulin degludec group compared with the insulin glargine group at the end of the trial, leading to similar rate of overall hypoglycemia between insulin glargine and degludec groups. Therefore, in daily clinical practice, adjustment of the premeal bolus insulin dose also needs be considered when switching to insulin degludec. The SMBG data in our study showed that postlunch blood glucose level was significantly lower after switching to insulin degludec injected at bedtime, suggesting that the peak action of insulin degludec occurs 14-15 hours after injection. Consistent with this finding, another study showed that the trough blood glucose was recorded at daytime when insulin degludec was injected at bedtime [16]. In addition, one review showed that the peak of the glucose-lowering effect of insulin degludec appeared about 12 hours after injection [17].

In addition to the longer duration of action of basal insulin, its effects on daily and day-to-day variability of plasma glucose should be noted. Heise et al. [8] reported that the use of insulin degludec resulted in lower day-to-day variability in blood glucose compared to insulin glargine in type 1 diabetes patients. However, different from this study, our results showed that switching to insulin degludec did not reduce MODD, an index of plasma glucose day-to-day variation, which was consistent with a previous study in Japan [13, 14]. The precise reason for the inconsistency with the overseas study remains unknown. Heise et al. [8] examined the glucose fluctuation by the glucose clamp method after very long fasting, which was not different from our method by CGM. Therefore, the inconsistency might be due to differences between experimental and real-world study.

Our study has certain limitations. Our study extended over a short period of time and included a limited sample size. In addition, the carry-over effect of HbA1c could not be completely excluded because our study was not a randomized controlled trial. Therefore, a crossover trial or randomized controlled trial of a larger sample size is needed in the future.

In conclusion, our study demonstrated that glycemic control 12 weeks after switching to once-daily insulin degludec injection with 20% dose reduction was comparable to that in patients treated with twice-daily injection of basal insulin injections and that such switching did not change the frequency of nocturnal hypoglycemia recorded by CGM.

## Figures and Tables

**Table 1:** Baseline characteristics of the subjects.

Age (years)	54.8 ± 14.5
Gender (male/female)	7/15
BMI (kg/m^2^)	22.1 ± 3.1
Diabetes duration (years)	14.6 ± 9.0
Kinds of basal insulin	
Insulin glargine	8
Insulin detemir	14
Fasting plasma glucose (mg/dL)	202.8 ± 81.4
Fasting C-peptide (ng/mL)	
Detection (*n* = 4)	0.153 ± 0.215
Below the detection limit (*n* = 18)	<0.015
Total daily dose/body weight (U/kg)	0.70 ± 0.21
Basal insulin/total daily insulin	0.38 ± 0.08
Complications (%)	
Retinopathy	31.8
Nephropathy	27.2
Neuropathy	63.6

Data are number/percentages of patients or mean ± SD.

**Table 2:** Effects of switching to insulin degludec on glycemic control.

	Baseline	4 weeks	12 weeks	*P* ^*∗*^
Fasting plasma glucose (mg/dL)	203.2 ± 81.2	165.5 ± 82.1	206.5 ± 122.4	0.91
HbA1c (%)	8.5 ± 1.4	8.6 ± 1.6	8.7 ± 1.6	0.28
Glycosylated albumin (%)	24.9 ± 5.0	25.3 ± 5.2	24.7 ± 3.6	0.81
Basal insulin dose (U)	15.2 ± 7.6	13.3 ± 6.8	11.6 ± 6.9	<0.01
Bolus insulin dose (U)	24.8 ± 11.1	24.9 ± 11.1	25.6 ± 11.4	0.08
Total daily dose (U)	40.0 ± 17.3	38.1 ± 16.6	37.9 ± 16.7	<0.01

Data are mean ± SD.

^*∗*^Between baseline and 12 weeks after switching to insulin degludec by the Mann-Whitney *U* test.

**Table 3:** Comparisons of glucose fluctuation and the frequency of hypoglycemic episodes over 4 days measured by iPro2 before and after switching from basal insulin to insulin degludec.

	Before	4 weeks	*P*
Daytime (6:00–23:59)			
Average blood glucose (mg/dL)	184.1 ± 45.8	189.6 ± 52.7	0.58
SD (mg/dL)	68.4 ± 14.5	66.5 ± 17.1	0.64
MODD (mg/dL)	72.1 ± 16.0	74.0 ± 23.0	0.68
Frequency of hypoglycemia (%)	6.1 ± 8.0	6.5 ± 9.7	0.87
Nighttime (0:00–5:59)			
Average blood glucose (mg/dL)	156.5 ± 63.7	174.6 ± 58.5	0.05
SD (mg/dL)	22.6 ± 10.9	24.8 ± 10.7	0.25
MODD (mg/dL)	66.2 ± 30.7	83.3 ± 36.9	0.08
Frequency of hypoglycemia (%)	14.4 ± 17.0	11.1 ± 15.0	0.45

Data are mean ± SD.

CGM data were analyzed for 4 consecutive days.
